# Addressing Vulvovaginal Atrophy (VVA)/Genitourinary Syndrome of Menopause (GSM) for Healthy Aging in Women

**DOI:** 10.3389/fendo.2019.00561

**Published:** 2019-08-21

**Authors:** Rossella E. Nappi, Ellis Martini, Laura Cucinella, Silvia Martella, Lara Tiranini, Alessandra Inzoli, Emanuela Brambilla, David Bosoni, Chiara Cassani, Barbara Gardella

**Affiliations:** ^1^Research Center for Reproductive Medicine, Gynecological Endocrinology and Menopause, University of Pavia, Pavia, Italy; ^2^Department of Clinical, Surgical, Diagnostic and Pediatric Sciences, University of Pavia, Pavia, Italy; ^3^Obstetrics and Gynecology Unit, IRCCS San Matteo Foundation, University of Pavia, Pavia, Italy

**Keywords:** vulvovaginal atrophy (VVA), genito-urinary syndrome of menopause (GSM), aging, longevity, vaginal dryness, dyspareunia, female sexual dysfunction (FSD), quality of life (QoL)

## Abstract

Vaginal health is an essential component of active and healthy aging in women at midlife and beyond. As a consequence of hormonal deprivation and senescence, the anatomy and function of urogenital tissues are significantly affected and vulvovaginal atrophy (VVA) may occur. In a high proportion of postmenopausal women, progressive and chronic VVA symptoms have a strong impact on sexual function and quality of life. The new definition of genitourinary syndrome of menopause (GSM) comprises genital symptoms (dryness, burning, itching, irritation, bleeding), sexual symptoms (dyspareunia and other sexual dysfunctions) and urinary symptoms (dysuria, frequency, urgency, recurrent urinary infections). Many variables (age, sexual activity and partnership status) influence the clinical impact VVA/GSM symptoms and attitudes of elderly women to consult for receiving effective treatments. Psychosocial factors play a critical role in sexual functioning, but the integrity of the urogenital system is as well important affecting many domains of postmenopausal women's health, including sexual function. Several international surveys have extensively documented the need to improve VVA/GSM management because of the strong impact on women's daily life and on couple's intimacy. Health care providers (HCPs) need to be proactive in the early recognition of VVA/GSM in order to preserve urogenital and sexual longevity, by using hormonal and non-hormonal strategies. The clinical diagnosis is based on genital examination to identify objective signs and on the use of subjective scales to rate most bothersome symptoms (MBS), especially vaginal dryness. Recent studies point to the importance of addressing VVA/GSM as a potential early marker of poor general health in analogy with vasomotor symptoms. Therefore, a standard of VVA/GSM care in elderly women is desirable to enhance physical, emotional and mental well-being.

## Introduction

Women live longer than men all around the world (1) and in developed countries they expect to survive more than 30 years following natural menopause, which usually occurs between 48 and 52 of age (2). That being so, the impact of reproductive aging on healthy longevity becomes increasingly important because of the potential conditions associated with menopause-related hormonal deficiency (3). Estrogen deprivation is the hallmark of ovarian exhaustion leading to the manifestation of several signs and symptoms with a significant impact on quality of life (QoL) and on physical, mental and sexual health (4). Even androgen insufficiency, an endocrine feature more evident in women with premature ovarian failure (natural, surgical, iatrogenic), may contribute to the clinical events related to menopause (5). Separating the effect of menopause from the variety of changes associated with senescence is quite difficult, but recent observations bring about the idea that menopause accelerates biological aging, especially when reproductive failure occurs prematurely (6).

The present narrative review points to the importance of addressing the chronic condition of vulvovaginal atrophy (VVA)/genitourinary syndrome of menopause (GSM) in the context of promoting urogenital and sexual longevity in women at midlife and beyond. It merely reflects the expert opinion of the authors by analyzing the amount of available evidence (1990–2019) in this complex field of research. Therapeutic strategies to effectively manage sexual symptoms associated with VVA/GSM have been reviewed extensively elsewhere (7–12) and, in here, they will be discussed briefly to serve the scope of preventing severe VVA/GSM in elderly women.

## Menopause and Urogenital Aging

Among the multitude of menopausal complaints, vasomotor symptoms (hot flushes and cold or night sweats) and vaginal dryness have clearly shown a strong relationship with low estrogens during and after the menopausal transition (13). Up to 80% of women experience vasomotor symptoms during menopause with an average duration of 10 years and a variable degree of severity (14). Untreated vasomotor symptoms may represent a biomarker of chronic postmenopausal conditions such as cardiovascular disorders and osteoporosis (15). However, they do not usually progress over time (16) and remain problematic for a lower number of postmenopausal women aged 60–65 years (17). Unlike vasomotor symptoms, vaginal dryness is highly present also in older women because it is the cardinal symptom of vulvovaginal atrophy (VVA) (18), a chronic condition starting around menopause, mainly as a consequence of estrogen deficiency (19), and progressing with chronological aging and medical morbidity (20). The majority of postmenopausal women have signs of VVA upon physical examination, especially if they consult for vaginal dryness (21), but less than half of the postmenopausal population report VVA symptoms as bothersome in international surveys (22–25). There is a lack of understanding surrounding vagina health (26) and elderly women do not discuss VVA symptoms so easily because sexual health is a sensitive topic (27). In addition, the condition is believed to be transient and part of the natural aging phenomena (28, 29). In the Vaginal Health: Insights, Views & Attitudes (VIVA) survey, 55% of women with vaginal discomfort reported experiencing symptoms for 3 years or longer and only a minority (4%) attributed their symptoms to vaginal atrophy (25). Age, attitudes toward menopause, sexual activity, chronic disorders, previous and/or current use of menopausal hormone therapy and other biopsychosocial determinants influence the level of distress associated with VVA symptoms and the rate of reporting female sexual dysfunction (FSD) (30, 31). General and sexual health of the partner, as well as the quality and duration of the relationship, are also very important and addressing age-related changes in both members of a couple may contribute to a better management of VVA and sexual dysfunctions (32).

Urogenital aging is an old problem, newly recognized, which can be highly prevented upon early recognition of signs and symptoms (33). Vaginal dryness, followed by dyspareunia, is the most common symptoms reported by postmenopausal women both in surveys (22) and in clinical studies (21, 34). In the REVIVE surveys conducted both in United States (US) (23) and in Europe (EU) (24) the onset of VVA symptoms has already been reported in the majority of women within the perimenopause/early postmenopause. Interestingly, in the AGATA study, which included a sample of Italian women asking for a routine gynecological examination, a clinical diagnosis of VVA displayed a prevalence ranging from 64.7 to 84.2%, starting from 1 to 6 years after menopause (35). It is essential that health care providers (HCPs) are proactive to uncover the topic of vaginal health because women who discuss VVA with HCPs are twice as likely to be current specific-treatment users (59.7% as compared to 22.7% who did not discuss VVA) (28). It is frequent to encounter a disconnection in education, communication, and information between HCPs and their menopausal patients (36). The WISDOM survey outlined that the comfort level of HCPs when prescribing VVA treatment is still suboptimal, in particular in case they are not gynecologists (37). Education of women, adequate training of HCPs and provision of communication tools in order to facilitate the “uncomfortable” dialogue are potential solutions to address the barriers currently impeding patient–clinician interactions around sexual health (38).

Basic counseling is the first step in the management of postmenopausal sexual dysfunctions (39) and a standard process of care developed by the International Society for the Study of Women's Sexual Health (ISSWSH) may provide guidance to HCPs to effectively recognize sexual concerns and problems in women (40).

## Vulvovaginal Atrophy (VVA) or Genitourinary Syndrome of Menopause (GSM): What is in These two Names?

In recent years, VVA has a new name, genitourinary syndrome of menopause (GSM), to underline the multitude of genital, sexual and urinary symptoms associated with the anatomical and functional changes of vulvo-vaginal tissues occurring with menopause and aging (41). A terminology consensus conference cosponsored by the North American Menopause Society (NAMS) and by ISSWSH was held in May 2013 to review the most relevant scientific literature in the field of postmenopausal urogenital and sexual health. Following a 2-day discussion, acknowledged experts agreed on the need of having a new term to describe more accurately the condition previously known as VVA. The choice of GSM was the result of many considerations, including the need of a term more acceptable in the medical and public arena to improve and increase communication, research, education and management of urogenital and sexual symptoms in postmenopausal women. The definition of syndrome is used to describe a collection of clinical signs and symptoms (genitourinary) correlated with each other, that do not have to be all present and related to a single identifiable pathogenesis, but occur in a particular circumstance (menopause). That being so, GSM is defined as “a collection of symptoms and signs associated with a decrease in estrogen and other sex steroids involving changes to the labia majora/minora, clitoris, vestibule/introitus, vagina, urethra, and bladder. The syndrome may include but is not limited to genital symptoms of dryness, burning, and irritation; sexual symptoms of lack of lubrication, discomfort or pain, and impaired function; and urinary symptoms of urgency, dysuria, and recurrent urinary tract infections (Table 1). Women may present with some or all of the signs and symptoms, which must be bothersome and should not be better accounted for by another diagnosis” (41).

**Table 1:** Most common subjective and objective symptoms to diagnose vulvovaginal atrophy (VVA)/genitourinary syndrome of menopause (GSM) in daily practice.

**Subjective symptoms (0 = none; 1 = mild; 2 = moderate; 3 = severe; not applicable = N/A symptoms related to sexual activity)**
Vaginal Dryness Dyspareunia Irritation/Burning/Itching Dysuria Bleeding with sexual activity
**Objective signs (clinical scale: 0 = normal; 1 = mild; 2 = moderate; 3 = severe)**
Elasticity Vaginal folds Fluid secretion Epithelial thickness Moisture Color of the tissues

VVA is strictly related to estrogen deficiency and is an integral part of GSM (10). However, the new definition GSM includes signs and symptoms that cannot be all reversed by estrogen replacement and may require different strategies according to their true etiology (42). As examples, vulvar dermatological conditions (43), vulvodynia (44), and pelvic floor dysfunction (45) have an increased prevalence in postmenopausal women, may co-occur with VVA, but have their own specific treatment protocols. At present, the majority of data were published with available questionnaires and scales validated to identify VVA-associated signs and symptoms and further studies are need to fully understand the multitude of disturbances included in the GSM definition. Recently, a novel patient-reported outcome measure exploring experiences of women with GSM was designed for use in both clinical care and research (46). The hope is to gain new insight into the biopsychosocial determinants of GSM in order to tailor evidence-based treatments for the individual woman across different stages of post reproductive lifespan.

## Physio-Pathological Aspects of VVA/GSM

Hormonal fluctuations driving the female reproductive life cycle highly modulate the functional anatomy of the uro-genital and pelvic tract. Early data showed that untreated postmenopausal women displaying <50 pg/ml of circulating estradiol suffer more from symptoms associated with VVA (47). A historical study (the only citation prior 1990) demonstrated that even endogenous androgens may play a role because objective signs of VVA were less evident in postmenopausal women with significantly higher mean levels of androgens (androstenedione and testosterone) and gonadotropins (particularly LH). These women were more sexually active (intercourse frequency, three or more times monthly) as opposed to the sexually inactive women (intercourse frequency, <10 times yearly) (48). Whether stronger sexual desire and responsiveness driven by androgens protected against VVA or, alternatively, androgens had a direct action on peripheral tissues was not established by the “the use it or lose it” theory. However, these data are in line with the evidence that both circulating estradiol and its androgen precursors (dehydroepiandrosterone/dehydroepiandrosteronesulphate [DHEA/DHEAS], androstenedione, testosterone), as well as their local metabolites, are vital to maintain normal structure and function of the vagina and surrounding uro-genital tissues (49). Indeed, the science of intracrinology supports the idea that the age-related decline of circulating DHEA translates into a local intracellular deficiency of both estrogens and androgens, significantly contributing to poor vaginal health (50). During reproductive life, the vagina, vulva, pelvic floor muscles, endopelvic fascia, urethra, and bladder trigone display a significant amount of estrogen receptors (ERs, both α and β), which decline with menopause and may be restored by the use of systemic and local estrogen treatment. ERs are mainly expressed in the epithelium and in stromal and muscle cells of the human vagina. Even androgen receptors (ARs) are largely expressed at multiple levels (mucosa, submucosa, stroma, smooth muscles, and vascular endothelium) and cross-talk with ERs, influencing neurovascular and neuromuscular function under different endocrine conditions (51). Estradiol controls a plethora of cellular pathways regulating growth and proliferation, barrier function and pathogen defense (52). The main consequence of lacking estrogen stimulation is the loss of tissue elasticity by inducing fusion and hyalinization of collagen fibers and fragmentation of elastin fibers. The mucosa of the vagina, introitus, and labia minora becomes thin and pale and appears less hydrated. The vaginal canal becomes shorter and narrow because the vaginal rugae, the epithelial folds that allow for distensibility, progressively disappear. In addition, there is significant reduction of vascular support leading to a decrease of the volume of vaginal transudate and of other glandular secretions (53). Both estrogens and androgens contribute to pelvic nerve-stimulated genital blood flow, tissue response to neurotransmitters and sensory threshold to stimuli (51). Over time, intercellular acid mucopolysaccharide and hyaluronic acid are significantly reduced in the dermal layer. Moreover, there is a progressive dominance of parabasal cells with fewer intermediate and superficial cells. This means the vaginal squamous epithelium is quite completely estrogen deprived. Therefore, it becomes friable with petechiae, ulcerations, and eventually bleeding after minimal trauma (54–61). A thinner vaginal epithelium is also associated with a significant reduction of glycogen which translates into a lower amount of lactobacilli causing an increase in vaginal pH (between 5.0 and 7.5). The subsequent decrease of vaginal hydrogen peroxide allows the growth of other pathogenic bacteria (staphylococci, group B streptococci, and coliforms) causing atrophic vaginitis, vaginal discharge and odor. Indeed, lactobacilli diversity and abundance significantly decreased following menopause (62) and the vaginal microbiota of women with mild or moderate atrophy had a distinct bacterial community state, which may predispose to develop vaginitis and other uro-genital infections (63).

The neurovascular and neuromuscular substrates of the pelvic area are also impaired because the vulva, as well as the pelvic floor and the urinary tract, manifest similar anatomical and functional changes (64–66). In particular, entry dyspareunia, irritation, burning and itching of external genitals may be the result of the stenosis of the vulvar introitus. Indeed, hymeneal carunculae and the vestibule display less elasticity and the urethral meatus appears prominent and more vulnerable to trauma. Several changes of the urinary system (reduced urethral closure pressure, reduced sensory threshold in the bladder, and, in some cases, increased risk of rUTIs) may be observed as a consequence of the thinning of the urinary epithelium and weakening of the surrounding tissue (53).

## Key-Elements of VVA/GSM Diagnosis

Clinical interviews and rating scales to score the most bothersome symptoms (MBS) (Table 1) are useful instruments to measure subjective symptoms and to identify risk factors for VVA/GSM. Objective diagnosis is confirmed by an accurate pelvic examination, including gentle inspection of the vulva, vestibule, vagina, and urethra in order to recognize the signs of VVA/GSM (Table 1) which can be rated on validated scales (67). The Vaginal Health Index Score is a clinical tool that, by evaluating 5 parameters (vaginal elasticity, vaginal secretions, pH, epithelial mucous membrane, vaginal hydration), allows to obtain a final score defining the degree of atrophy in the genitourinary tract by assigning a single score to each parameter. Total score ranges from 5 to 25, with lower scores corresponding to greater urogenital atrophy (68). Vulva Health Index evaluates labia, urethra, clitoris, introitus as well as elasticity and pain during intercourse; total score ranges from 0 to 24, with higher scores corresponding with greater vulvar atrophy. If the Vulva Health Index is over 8 or there is score of 3 (severe) in any category, vulvar atrophy is suggested (69). In the most severe cases, tissues may be easily traumatized and irritated by touching or inserting the speculum (70). Organ prolapse or hypertonicity of the pelvic floor with secondary vaginism may be also present, as well as vulvovaginal signs which require a differential diagnosis by performing colposcopy or carrying out bacteriological analyses (11). In general, VVA/GSM is typically a clinical diagnosis and few laboratory tests may be used to support the evidence. Among them, the evaluation of vaginal pH and the vaginal maturation index (VMI) are the most used (41). With the VMI it is possible to identify the relative proportion of parabasal, intermediate, and superficial vaginal epithelial cells. Hypoestrogenism and atrophy are suggested when there is a dominance of parabasal cells, calculated on specimens obtained directly from the lateral upper vaginal walls. Thus, the shift to a higher number of superficial cells is a primary end-point of any treatments prescribed to relieve symptoms of VVA (71). Even, vaginal pH alone is a simple outpatient procedure, influenced by infections and intimate products, which reflects the hormonal milieu and its effects on the vaginal epithelium. Indeed, it consistently correlated with parabasal and superficial cells and the visual vaginal epithelial changes and symptoms of dryness and dyspareunia (72).

In both clinical and research settings, subjective assessment (the MBS approach) and objective assessments of VVA (measurement of vaginal maturation index and vaginal pH) should be combined according to a recent systematic literature search (73). Even though a high rate of subjective symptoms is associated with a clinical diagnosis of VVA/GSM in over 90% of the cases (21), objective signs and subjective symptoms have a different prevalence distribution in the years after menopause and are not strictly associated (35). However, self-reported and visible vaginal dryness do correlate and together with ph> 5, mucosal pallor, and rugae thinning seem to be the most important objective signs to make a diagnosis (35). On the other hand, the presence of other vulvar and urinary signs are relevant to the severity of VVS/GSM and its impact on women's daily living (74).

**Figure 1:**
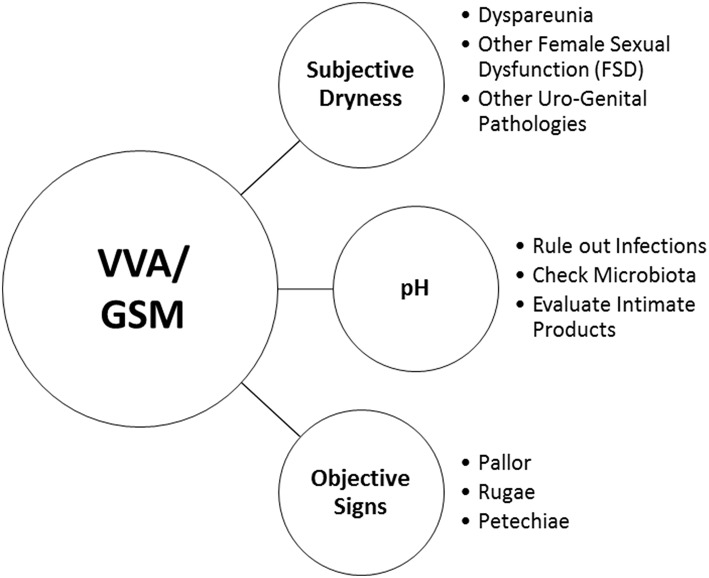
A very simple check-list to diagnose VVA/GSM in routine clinical practice.

Notwithstanding these findings, HCPs may pose very simple questions to facilitate an open conversation on urogenital health and to record the variety of vaginal, vulvar and urinary symptoms. Visual vaginal, vulvar and pelvic assessment by HCPs is a useful measure for diagnosing VVA/GSM and assessing response to treatment. Moreover, it may help HCPs to identify women at risk of vaginal dryness and dyspareunia, and allow them to proactively engage in conversations about sexual health (75). Figure 1 reports a very simple check-list to diagnose VVA/GSM in routine clinical practice.

Women with breast cancer and other gynecological malignancies are at very high risk of VVA and associated symptoms. Indeed, endocrine chemotherapy, surgery and/or radiation may induce profound changes at urogenital levels which have to be timely recognized in the oncologic care (76, 77). Moreover, we lack data on VVA/GSM in women with spontaneous premature ovarian insufficiency, even though it is likely that the condition is more distressing due to the younger age of these patients (78). Older women and those who abstain from sexual activity may suffer even more of VVA/GSM with vaginal and introital stenosis, fusion of the labia minora to the labia majora, and other urogenital conditions (79). Preventive gynecology is significantly challenged by the presence of severe VVA/GSM. Indeed, it may be difficult to adequately assess both cytologic and colposcopic findings to prevent cervical cancer. On the other hand, an episode of postmenopausal bleeding, very common in women with VVA/GSM, may cause an urgent referral to exclude endometrial cancer and other malignancies. Finally, even if less common, an early diagnosis of cancer may be delayed by vaginal synechiae and hematocolpos due to vaginal occlusion (80–82).

## The Burden of VVA/GSM on Women's Sexual Function and Quality of Life (QoL)

In the last decade, many international surveys attempted to clarify the impact of VVA/GSM on sexual function and QoL (Table 2) indicating that a proactive approach to conversations about vulvovaginal discomfort would improve diagnosis and treatment (22). Even though the proportion of women who are sexually active decreases with advancing age, the value of discussions about sexual health is still high in elderly women who are in partnership (83). In the survey of Midlife Development in the United States (MIDUS II) women who were married or cohabitating had approximately 8 times higher odds of being sexually active, with more than 30% of women over 65 years reporting sexual activity at least once a week (84). Sexual satisfaction is highly dependent on many psychosocial aspects related to well-being (85). In addition, dimensions of sexual response are part of the domino effect of menopausal symptoms, including weight gain, depression, anxiety and poor physical health (86). VVA/GSM is a clear medical condition that can be associated with impairment of sexual activity and intimacy within couples at menopause (19). VVA symptoms have an approximately linear relationship with sexual functioning (87) and VVA correlates with sexual inactivity in the Hormone Therapy (HT) Trials of the Women's Health Initiative (WHI) (88). These findings are in contrast with an early study showing in a little sample of pre- and postmenopausal women that current sexual activity was not associated with differences in vaginal length or introital caliber (89). On the other hand, the international CLOSER survey investigated the impact of VVA on postmenopausal women and on male partners demonstrating that intimacy avoidance was attributed to painful sex by a significant proportion of women (55%) and men (61%) (90). That being so, the assessment of sexual well-being at menopause should rule out not only the clinical signs of VVA/GSM but also the multitude of aspects associated with it, especially hypoactive sexual desire disorder (HSDD) which is a strong determinant of maintaining sexual activity and emotional intimacy within the relationship (91).

**Table 2:** Most common dimensions affected by vulvovaginal atrophy (VVA)/genitourinary syndrome of menopause (GSM) in international surveys.

**Sexual dimensions**
Satisfaction Intimacy Spontaneity Loving relationship Sexual activity
**Quality of life dimensions**
Sleep Enjoinment Sportive activity Work/social activity Feminine role Sense of youth

Cultural aspects are strongly related to the interpretation of results from surveys on VVA/GSM and explain differences in reporting bothersome symptoms and consequences associated with them. For example, women reporting VVA in Southern Europe stopped having sex in 18 % of the cases (92), in Northern Europe in 22% (92), in UK in 27% (93) and in North America (US and Canada) in 29% (94). In addition, both US and EU REVIVE surveys underlined the strong impact of VVA on sexual satisfaction and sexual spontaneity, as well as on intimacy and relationship with the partner (23, 24). Of interest, EU participants acknowledged a significantly higher impact of VVA symptoms on sexual intercourse and partner interaction than US participants, and both cohorts were observed to have differences between their respective VVA symptom profiles (95). Apart cultural attitudes in the health care system or in the importance to maintain sexual activity over time, other elements of difference may be found between US and EU samples at baseline, including age, marital status, education, and working activity (95). Indeed, other studies indicate that the true prevalence of each symptom and the rate of distress associated with it are significantly influenced by many factors, namely age and sexual activity (96, 97). Dyspareunia is generally less reported later in life mainly because older women are less likely to still have a spousal or other intimate relationship (83). Behavioral profiles of postmenopausal women play also a role in disclosing VVA symptomatology and actively seeking treatment (98). Data collected in the CLOSER survey indicated that the VVA condition related to many dimensions of womanhood, in particular perception of aging and poor health (90, 99). In the “women's voices in the menopause” survey (27), 52% of respondents with vaginal discomfort reported an impact on their QoL. Both VIVA and CLOSER international surveys further explored the dimension affected by self-reported VVA symptoms demonstrating an influence on working, social activity and other aspects of personal well-being (24, 25). In addition, other data indicated that VVA is associated with a clinically significant impact on QoL that may be comparable to that seen in serious conditions such as arthritis, chronic obstructive pulmonary disease, asthma and irritable bowel syndrome (100, 101).

The EVES study collected very accurate information in a clinical population of EU (Italy and Spain) postmenopausal women aged 45–75 years reporting at least one subjective VVA symptom and objectively diagnosed with VVA during gynecological examination. Women scored 19 potentially VVA-related complaints on a 4-point severity scale (absent, mild, moderate and severe) and filled in both the EuroQol questionnaire (EQ-5D-3L) (102) and the Day-to-Day Impact of Vaginal Aging (DIVA) questionnaire to measure the impact of VVA on several dimensions of QoL (103). Sexual function and distress were also evaluated by validated questionnaires (104, 105). During gynecological clinical assessment, signs of VVA were rated in order to calculate the Vaginal Health Index (68) and the Vulva Health Index (69). The main outcomes of EVES showed that of a total of 2,160 evaluable women, 66.3, 30.5, and 11.2% suffered from severe vaginal, vulvar, and urinary symptoms, respectively. VVA was confirmed in more than 90% of the participants. Both generic and vaginal aging-related QoL scores showed a significant relationship with the different types of severe VVA symptoms. QoL questionnaires displayed worse scores in women where the diagnosis of VVA was confirmed by gynecologic examination. The severity of urinary symptoms showed a more strong impact on all DIVA components (daily activities, emotional well-being, sexual functioning and self-concept/body image) compared to vaginal and vulvar symptoms (74). This data confirmed recently reported observations on predictors of impact of vaginal symptoms, in which women with urinary incontinence reported a higher impact of VVA symptoms on three of the four DIVA dimensions (not sexual functioning) (106). In the Italian subset of 1,226 postmenopausal women, those with objective confirmation of VVA had worsened sexual function and distress when compared with the patients having only subjective VVA symptoms (107). Interestingly enough, postmenopausal women with VVA receiving treatment complained of more severe symptoms than those untreated. Moreover, time since menopause was significantly higher in women treated for VVA. Collectively, EVES data indicate that VVA treatments should ideally be initiated at a younger age when symptoms commence and cause distress, before the condition becomes very severe and difficult to be reverted (108).

## General Principles for VVA/GSM Treatment

The chronic nature of VVA/GSM indicates that effective treatments should preferably be prescribed at the onset of the symptoms and signs of atrophic changes of the vagina, early before severe pictures of the condition occur, and should be continued over time in order to maintain their benefits (109). The therapeutic approach needs to be personalized and women's preferences have to be taken into account because the level of comfort with a given therapy is strongly influenced by a multitude of individual and socio-environmental factors (110). Apart the embarrassment to discuss an intimate condition, fears of hormones are a major barrier (24), in spite of the very reassuring safety data obtained with local estrogen therapy (LET) (111), the first-line hormonal treatment for VVA/GSM according to guidelines of menopausal scientific societies (53, 112, 113). Various local estrogen treatments are equally effecting in reversing VVA/GSM symptoms, including dyspareunia and other associated sexual dysfunction, alone or even combined with systemic HT. With low-dose LET, systemic estrogen absorption is minimal, and serum estradiol levels remain in the postmenopausal range permitting the use in women with or at high risk for breast cancer, after a discussion of risks and benefits and review with oncologists (76). Local androgens, such as DHEA pessaries and testosterone cream, are new therapeutic options that await for further confirmation (49). Another option approved by Medical Authorities is ospemifene, a third-generation selective estrogen receptor modulator, which is an oral medication for the treatment of VVA associated symptoms (114). It is currently indicated for women, who are not candidates for LET or whenever other treatments, including LET, were not effective to relieve vaginal dryness and dyspareunia (15).

Non-hormonal strategies may be used in women of any age in which hormonal treatments are contraindicated or co-treating women prescribed with systemic/vaginal hormone therapy. The prescription of vaginal moisturizers and lubricants and the maintenance of sexual activity may be helpful in improving vaginal dryness-related symptoms. However, a few clinical trials have been performed to assess the efficacy of such products. Lubricants are short-acting substances (water-, silicone-, or oil-based) which are useful to reduce friction during sexual activity, whereas moisturizers are longer acting than lubricants and may exert a trophic effect (115). Pelvic floor muscle training (PFMT) program in postmenopausal women with urinary incontinence is feasible and improves VVA/GSM symptoms and signs, as well as displays a positive impact on activities of daily living, QoL and sexual function (116). Microablative fractional CO_2_ laser, the non-ablative vaginal Erbium YAG laser (VEL) and energy-based devices are increasingly used to alleviate VVA/GSM symptoms with promising results and a good safety profile (117).

## VVA/GSM as a Negative Marker of Women's Aging: Is There Enough Evidence?

Urogenital and sexual longevity is an integral part of healthy aging in postmenopausal women and their partners. The severity of VVA/GSM and the type of prevailing symptoms are mostly influenced by the multitude of clinical phenotypes of postmenopausal women depending on a wide range of biopsychosocial variables which are difficult to estimate in large scale trials. It is known that women loose less years of sexually active life because of poor health than men (118). This data confirm the multidimensional nature of women's sexuality with psychosocial factors (relationship satisfaction, communication with romantic partner, and importance of sex) mattering more than biological aging to sexual satisfaction among midlife and older women (84). That being so, the presence of severe VVA/GSM cannot be considered a negative marker of general health as it had been demonstrated for erectile dysfunction in aging males (119). However, coital sexual activity is associated with an excellent or very good general health also in women, as it is in men (83), and it is certainly influenced by a healthy genital response. Even if it has been difficult to establish a clear link between cardiovascular and metabolic health and women's sexual dysfunctions (120), there is no doubt that several chronic conditions may be associated with poor sexual functioning (121). It is fascinating to speculate on the evidence that vaginal dryness is the only other symptom very sensitive to estrogen deprivation apart hot-flushes (13). Given the clear association of vasomotor symptoms with negative long-term health consequences across aging (15), we cannot exclude that even severe VVA/GSM may represent an early marker of poor general health, a hypothesis that needs further exploration by investigating objective parameters of such chronic condition in relationship with other aspects of women's well-being. Interestingly, baseline characteristics and medical history were tabulated for a VVA cohort identified from two US administrative claims databases (9,080 women aged 40–79 years) and matched controls without VVA. The mean age at baseline was 60.2 years for both but the Deyo-Charlson comorbidity index was significantly higher, with a significantly higher proportion of women in the VVA cohort with a diagnosis of angina, osteoporosis, migraines, insomnia, or anxiety. As expected VVA patients had a significantly higher incidence of each of six genitourinary conditions (“urinary tract infections,” “other/unspecified genitourinary symptoms,” “other inflammatory diseases of female pelvic organs,” “menopausal disorders,” “female genital pain and other symptoms,” and “other/unspecified female genital disorders”) compared to controls (122).

## Conclusions

The management of VVA/GSM is increasingly important in light of the feminilization of aging. Postmenopausal women are becoming aware that preserving urogenital and sexual longevity is a major step in gender equality and healthy living. HCPs should address the issue in daily clinical practice with the aim to prevent the long-term health consequences associated with estrogen deprivation (123). Early recognition of signs and symptoms of VVA/GSM, individual counseling and personalized treatment strategies are key-steps in helping women to maintain QoL.

## Author Contributions

RN: conception and design. EM, LC, LT, AI, EB, SM, and DB: acquisition, analysis, and interpretation of data. CC and BG: drafting the article. RN and BG: revising for intellectual content. RN, EM, LC, SM, LT, AI, DB, CC, and BG: final approval.

### Conflict of Interest Statement

During the past 2 years, RN had a financial relationship (lecturer, member of advisory boards and/or consultant) with Bayer HealthCare, Endoceutics, Exceltis, Gedeon Richter, MSD, Novo Nordisk, Palatin, Pfizer, Shionogi, Teva, and Theramex. These companies have no involvement with the study. The remaining authors declare that the research was conducted in the absence of any commercial or financial relationships that could be construed as a potential conflict of interest.
